# The EBV-Encoded Oncoprotein, LMP1, Recruits and Transforms Fibroblasts via an ERK-MAPK-Dependent Mechanism

**DOI:** 10.3390/pathogens10080982

**Published:** 2021-08-03

**Authors:** Alexandra M Davis, Abigail Rapley, Christopher W Dawson, Lawrence S Young, Mhairi A Morris

**Affiliations:** 1 Faculty of Health and Life Sciences, De Montfort University, Leicester LE1 9BH, UK; Alexandra.davis2@dmu.ac.uk (A.M.D.); abigail.rapley@icloud.com (A.R.); 2 Warwick Medical School, University of Warwick, Coventry CV4 8UW, UK; C.Dawson.3@warwick.ac.uk (C.W.D.); L.S.Young@warwick.ac.uk (L.S.Y.); 3 School of Sport, Exercise and Health Sciences, Loughborough University, Loughborough LE11 3TU, UK

**Keywords:** LMP1, EBV, NPC, fibroblast, myofibroblast, cancer-associated fibroblasts, ERK-MAPK, tumour microenvironment, tumour stroma

## Abstract

Latent membrane protein 1 (LMP1), the major oncoprotein encoded by Epstein–Barr virus (EBV), is expressed at widely variable levels in undifferentiated nasopharyngeal carcinoma (NPC) biopsies, fueling intense debate in the field as to the importance of this oncogenic protein in disease pathogenesis. LMP1-positive NPCs are reportedly more aggressive, and in a similar vein, the presence of cancer-associated fibroblasts (CAFs) surrounding “nests” of tumour cells in NPC serve as indicators of poor prognosis. However, there is currently no evidence linking LMP1 expression and the presence of CAFs in NPC. In this study, we demonstrate the ability of LMP1 to recruit fibroblasts in vitro in an ERK-MAPK-dependent mechanism, along with enhanced viability, invasiveness and transformation to a myofibroblast-like phenotype. Taken together, these findings support a putative role for LMP1 in recruiting CAFs to the tumour microenvironment in NPC, ultimately contributing to metastatic disease.

## 1. Introduction

Epstein–Barr virus (EBV) was the first human DNA tumour virus to be identified in the 1960s and is estimated to infect 90–95% of the global population. Although typically asymptomatic, infection with EBV in discrete geographical populations carries a higher-than-average risk of developing certain cancers of B cell and epithelial cell origin. Amongst these virus-associated cancers is nasopharyngeal carcinoma (NPC), a type of head and neck cancer that, whilst relatively rare globally (1.2 cases per 100,000 in the global population), exhibits high incidence rates in parts of Southeast Asia, with between 15–50 cases per 100,000 in the population [1]. NPC is linked with multiple aetiological factors, including dietary and environmental risk factors, genetic predisposition, and of course, infection with EBV [2].

EBV encodes 9 latent gene products, amongst which is latent membrane protein 1 (LMP1), considered to be the major oncoprotein of EBV due to its ability to transform B cells and rat fibroblasts in vitro [3], as well as its ability to induce an epithelial-to-mesenchymal transition (EMT) in epithelial cells in vitro [4,5,6,7]. LMP1 acts as a constitutively active tumour necrosis factor (TNF) receptor, activating a plethora of mitogenic signalling pathways that are frequently dysregulated in many human cancers, including both the canonical and non-canonical NF-κB pathways, the mitogenic ERK-MAPK and p38-MAPK pathways, the pro-survival PI3K/Akt pathway, the stress-related JNK/SAPK pathway, as well as the Smad-independent activin A/TGFβ signalling cascades, which may contribute to the fibrotic response that drives carcinoma growth and metastases [8,9].

The exact role of LMP1 in the pathogenesis in NPC remains incompletely understood since expression levels of the oncoprotein in NPC biopsies varies so widely [10]; however, it is believed to play an important role during the early stages of the disease process, in both the malignant transformation of epithelial cells in the nasopharynx and also the recruitment and activation of the characteristic inflammatory infiltrate [11].

There is increasing attention being paid towards the role of the tumour microenvironment (TME) in driving cancer cell growth and metastases in EBV-associated cancers [12]. Whilst NPC is well-known for its characteristic infiltrate of tumour-specific T cells, it is often also heavily infiltrated by other immune cell types too, including B cells and natural killer (NK) cells, as well as non-immune cells, such as mesenchymal stem cell-derived fibroblasts [13].

Tumours have been described as “wounds that fail to heal” [14] since there are many shared similarities between the wound healing process and tumourigenesis, including the recruitment and activation of fibroblasts to myofibroblasts or cancer-associated fibroblasts (CAFs) and ECM deposition [15]. CAFs in the TME display an altered immunophenotype compared with their normal quiescent counterparts, and in NPC, CAFs tend to surround “nests” of tumour cells whereby their presence can serve as indicators of poor prognosis [16,17]. More recently, LMP1 packaged in extracellular vesicles has been shown to activate normal fibroblasts into CAFs [18], and can enhance cancer progression in NPC [19].

In addition to their role in secreting proteases that degrade the extracellular matrix (ECM) surrounding a tumour to facilitate metastasis [20,21], CAFs also provide support to cancer cells in the form of paracrine cytokine release to fuel their insatiable growth [22,23,24]. CAFs form heterologous cell–cell interactions with cancer cells to activate integrin-mediated outside-in pro-survival signalling pathways that contribute to chemoresistance [25,26]. CAFs also recruit other stromal cell types, such as mesenchymal stem cells (MSCs), with immunomodulatory properties [27]. Indeed, some studies indicate that bone marrow-derived MSCs may, in fact, be a source of CAFs themselves [28,29].

In previous studies, we have demonstrated a role for LMP1 in the secretion and deposition of fibronectin [9], enhanced cell migration [30], and the induction of an EMT phenotype in epithelial cells [7]. More recently, this EMT phenomenon in LMP1-expressing cells has been attributed to neurotrophic tyrosine kinase receptor type 2 (NTRK2)-mediated Akt/ERK signalling, culminating in anoikis resistance [31]. While little is known about the role of LMP1 in the generation of CAFs in NPC [32], in this current study, we demonstrate a role for LMP1 in the recruitment of fibroblasts via an ERK-MAPK-dependent mechanism, with a partial contribution from TGFβ, enhanced fibroblast proliferation, and the subsequent transformation of fibroblasts to a myofibroblast-like phenotype in vitro. Moreover, we provide evidence that LMP1-expressing cells may secrete a factor, or factors, that can also transform epithelial cells to adopt a mesenchymal phenotype, which may serve as another potential source of CAFs in the NPC tumour microenvironment.

## 2. Results

### 2.1. Conditioned Medium from LMP1-Expressing Cells Enhances Fibroblast Motility

Despite the paucity of research into the origin and function of CAFs in NPC, there is emerging evidence to suggest that CAFs play a key role in disease pathogenesis [16,17]. Even less is known about the interaction between LMP1 and the recruitment and activation of CAFs in NPC. However, given the numerous synergies between the mechanisms involved in CAF-mediated tumourigenesis and the signalling pathways engaged by LMP1, the idea that LMP1 may be important in driving CAF formation deserves closer inspection [32].

First, we sought to establish whether LMP1 could alter fibroblast migration and invasion—a key step in the recruitment of local fibroblasts to the TME prior to their transformation. The migration rate of adult human dermal fibroblasts (HDFa) was measured using the wound healing scratch assay (WHSA), and invasion was measured using Transwell assays. Both assays were conducted in the presence of conditioned medium from MDCK-pLNSX control and MDCK-LMP1-expressing cells or serum-free medium to control for cell-derived factors.

**Figure 1:**
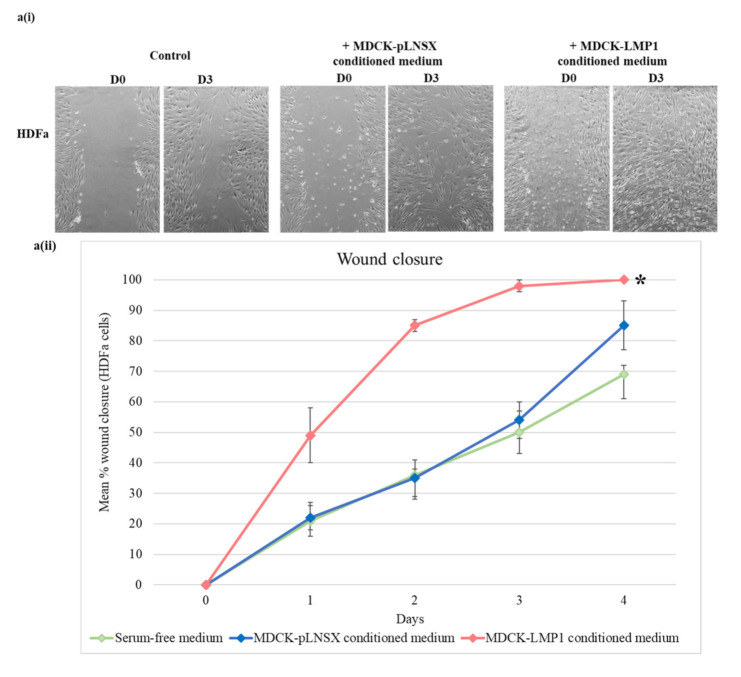

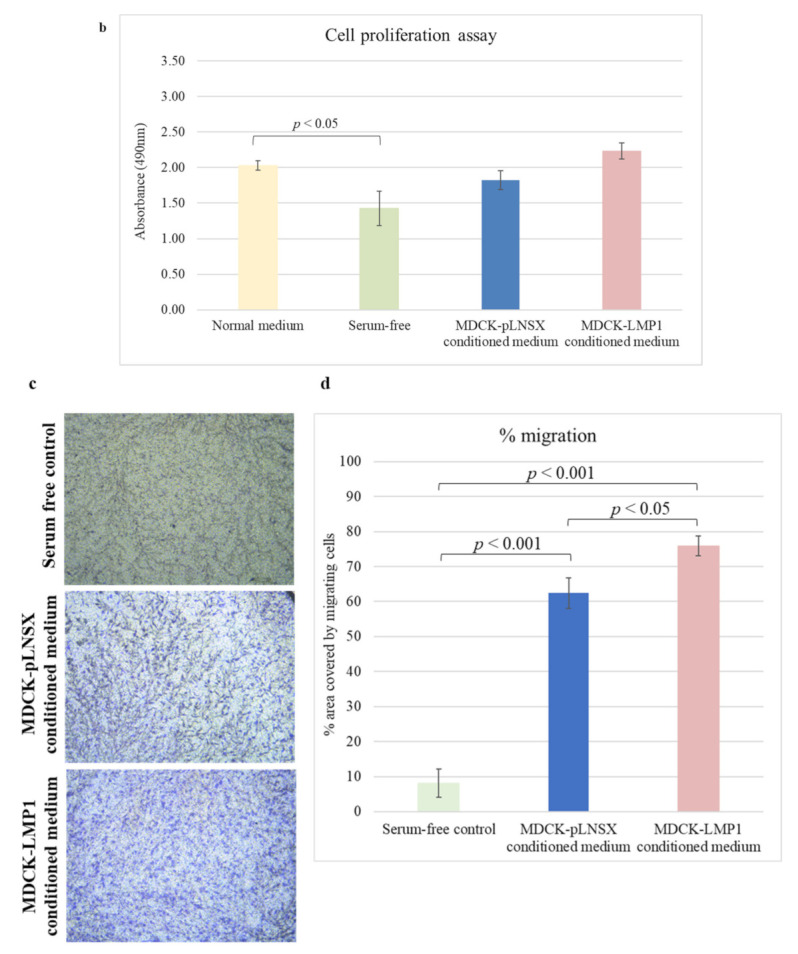
Conditioned medium from LMP1-expressing cells enhances the rate of fibroblast motility. (**a**(**i**)) The wound healing scratch assay confirms HDFa cell migration is enhanced by overnight culture in conditioned medium taken from LMP1-expressing MDCK cells. Images captured by phase contrast on the EVOS FL digital fluorescence microscope. Results are representative of experiments performed in triplicate. (**a**(**ii**)) ImageJ analysis quantified the wound area at set time points. The results depict mean % wound closure over time after scratch (mean ± SD, *n* = 3). Asterisk indicates result significantly different from the serum-free control * = *p* < 0.0005. Significant differences were determined using a mixed ANOVA. (**b**) The Promega CellTiter 96^®^ Aqueous One Solution Cell Proliferation Assay kit confirms the observed effects in (**a**) arise from enhanced motility and not enhanced proliferation. Results show absorbance after 3 days (mean ± SD, *n* = 3). Significant differences were determined using a one-way ANOVA. (**c**) Transwell migration assay further corroborates HDFa recruitment in response to LMP1-conditioned medium. Images were captured using an inverted Leica microscope, with attached Leica MC170 HD camera, and Leica Application Suite (LAS) software). Results are representative of experiments performed in triplicate. (**d**) ImageJ analysis determined percentage migration. Three representative fields per condition were measured and mean was calculated (mean ± SD, *n* = 3). Significant differences were determined using the Student *t*-test.

Representative images shown in Figure 1(ai,aii) demonstrate that conditioned medium from LMP1-expressing cells enhanced the rate of wound closure in HDFa cells within a 3-day time period. Statistical analysis by mixed ANOVA demonstrated a significant difference between all three conditions (F(8,96) = 13.993, *p* < 0.0005, partial η^2^ = 538). Further post hoc analysis revealed that conditioned medium taken from LMP1-expressing cells significantly enhanced the rate of wound closure compared to serum-free medium (*p* < 0.0005), whilst conditioned medium from control cells failed to have a marked impact on HDFa motility (*p* ≥ 0.05). In order to confirm that these effects were due to enhanced cell motility and not enhanced cellular proliferation, the CellTiter96^®^ Aqueous One Solution Cell Proliferation Assay kit was used (Figure 1b). Although there was a slight increase in proliferation across the 3 days in the cells treated with conditioned medium from LMP1-expressing cells when compared with that from both normal medium alone and medium from control cells, statistical analysis by one-way ANOVA demonstrated no significant change in levels of proliferation in response to treatment with conditioned medium taken from either cell line when compared with normal medium (*p* ≥ 0.05). However, there was a significant decrease in cellular proliferation in response to incubation in serum-free medium (*p* < 0.05).

Representative transwell images shown in Figure 1c demonstrate that HDFa cell recruitment is enhanced by conditioned medium taken from LMP1-expressing cells and to some extent by conditioned medium taken from control cells. Statistical analysis using the Student t-test confirmed that both control and LMP1-conditioned medium significantly affected HDFa recruitment (*p* < 0.001). While both conditions had a very strong significant effect, further statistical analysis between the two conditions demonstrated that this effect was more marked with medium from LMP1-expressing cells (Figure 1d); *p* < 0.05).

### 2.2. Conditioned Medium from LMP1-Expressing Cells Recruits Fibroblasts via a Chemotactic Gradient

**Figure 2:**
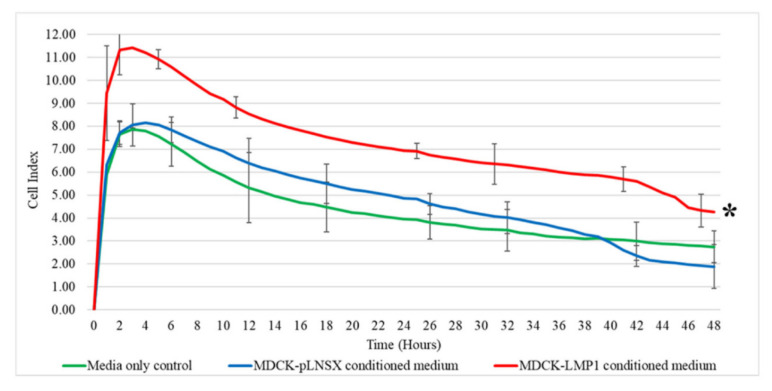
LMP1 recruits fibroblasts via a chemotactic gradient. Cell invasion and migration analysis using the RCTA-DP-xCELLigence System with CIM plates confirmed conditioned medium taken from LMP1-expressing cells enhanced the recruitment of fibroblasts within 48 h. Asterisk indicates result significantly different from the media only control (*p* < 0.001). Significant differences were determined using a mixed ANOVA (*n* = 3).

To assess whether the enhanced migration observed in Figure 1 was meaningful, we used an electrical impedance-based assay on the RTCA-DP xCELLigence System with CIM plates to investigate the ability of LMP1 to recruit fibroblasts via a chemotactic gradient. In brief, conditioned medium from cultures of either MDCK-pLNSX control cells or MDCK-LMP1-expressing cells was added to the bottom well of the CIM plates prior to seeding human dermal fibroblasts (HDFas) into the upper chamber. As shown in Figure 2, conditioned medium from LMP1-expressing cells significantly increased the rate of HDFa migration across the gold-plated sensors in the CIM plate (*p* < 0.001), while conditioned medium from control cells failed to have a marked impact on HDFa motility (*p* ≥ 0.05).

### 2.3. LMP1-Mediated HDFa Motility and Recruitment Requires ERK-MAPK and TGFβ Signalling

LMP1 has previously been reported to promote epithelial cell motility and invasion by engaging a number of signalling pathways, including ERK-MAPK [30,33,34]. LMP1 has also been shown to elevate TGFβ1 secretion in epithelial cells [9]. Well-established data supports the role of TGFβ in enhancing cell motility and invasion [35,36,37]. Therefore, we next sought to investigate further the role of these two signalling pathways in LMP1-induced fibroblast motility.

**Figure 3:**
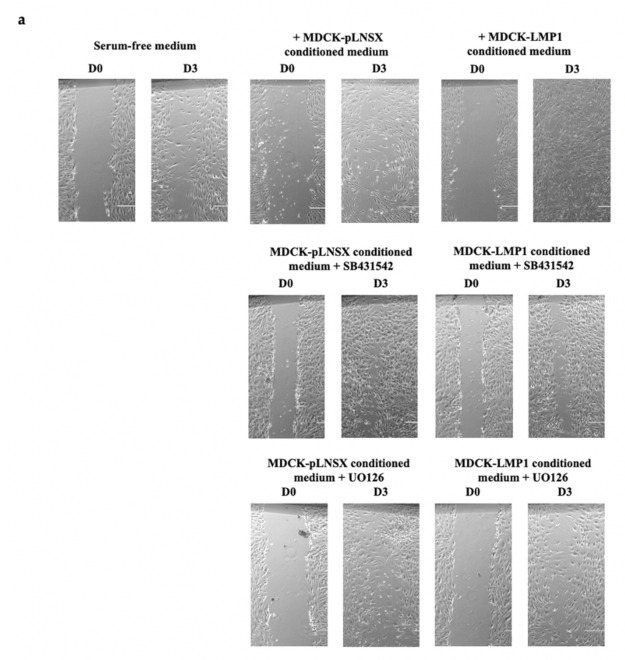

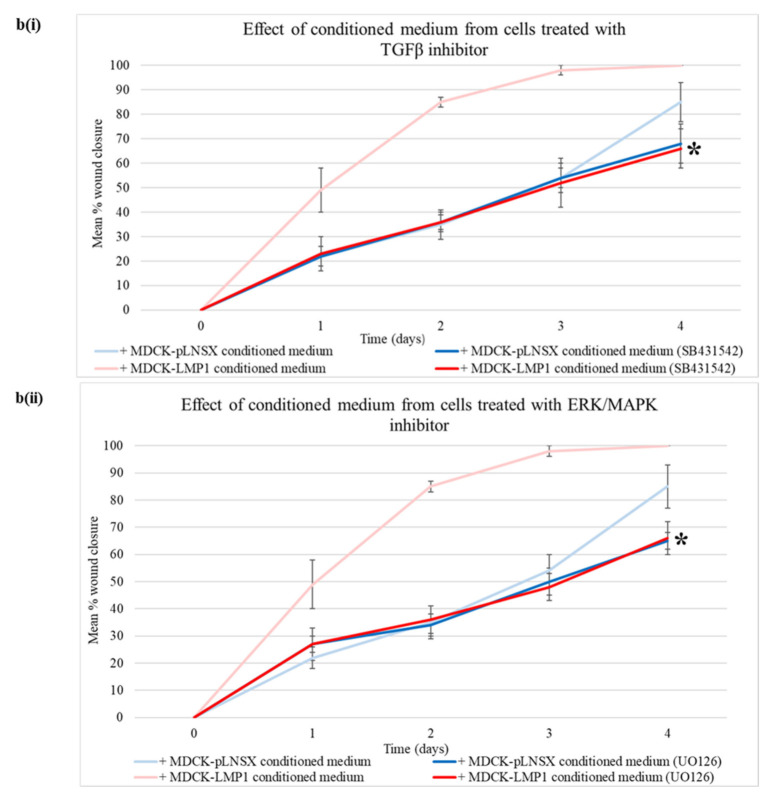
LMP1-mediated enhancement of HDFa motility is diminished by inhibition of TGFβ and ERK-MAPK signalling. (**a**) The wound healing scratch assay (WHSA) confirms the LMP1-mediated enhanced migratory ability of HDFa cells is diminished upon inhibition of both the ERK-MAPK, and to a lesser extent, the TGFβ signalling pathways. Images captured by phase contrast on the EVOS FL digital fluorescence microscope. Results are representative of experiments performed in triplicate. Bar = 1000 μm. (**b**) ImageJ analysis quantified the wound area at set time points. The results depict mean % wound closure over time after scratch (mean ± SD, *n* = 3). Asterisk indicates significant difference between results in the presence of each inhibitor compared to the absence of each inhibitor * = *p* < 0.0005. Significant differences were determined using a mixed ANOVA. (**i**) Fibroblasts treated with conditioned medium from control and LMP1-expressing cells in the presence and absence of the TGFβ type II receptor inhibitor, SB431542. (**ii**) Fibroblasts treated with conditioned medium from control and LMP1-expressing cells in the presence and absence of the ERK-MAPK inhibitor, UO126.

Using the WHSA, representative images shown in Figure 3a demonstrate that inhibition of both TGFβ and ERK-MAPK signalling using the small molecule inhibitors SB431542 and UO126, respectively, diminishes the rate of LMP1-mediated wound closure and ultimately cell motility and invasion in HDFa cells. Statistical analysis by mixed ANOVA confirmed a very strong significant difference in the rate of wound closure between cells treated with conditioned medium taken from LMP1-expressing cells cultured in the presence of both inhibitors compared to conditioned medium taken from LMP1-expressing cells cultured in the absence of both inhibitors (*p* < 0.0005; Figure 3(bi,bii)). No significant difference was found for the rate of wound closure between conditioned medium taken from control cells in the presence or absence of either inhibitor (*p* ≥ 0.05).

**Figure 4:**
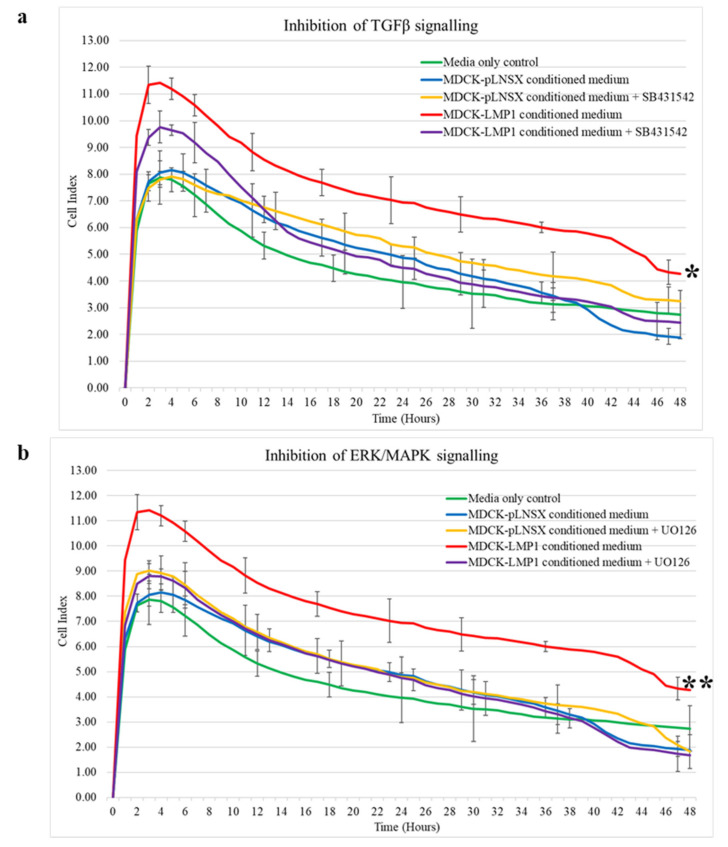
LMP1-mediated enhancement of HDFa recruitment and invasion is diminished by inhibition of TGFβ and ERK-MAPK signalling. Cell invasion and migration analysis using the RCTA-DP-xCELLigence System with CIM plates confirmed that conditioned medium from LMP1-expressing cells which enhanced the recruitment of fibroblasts is diminished by inhibition of both the ERK-MAPK, and to a lesser extent, the TGFβ signalling pathways within 48 h. (**a**) Inhibition of TGFβ signalling using the type I receptor inhibitor, SB431542. (**b**) Inhibition of ERK-MAPK signalling using the MEK1/2 inhibitor, UO126. Asterisk indicates results significantly different between the presence and absence of the inhibitor * = *p* < 0.01, ** = *p* < 0.001. Significant differences determined by mixed ANOVA (*n* = 3).

Further validation was performed using the RTCA-DP-xCELLigence System with CIM plates whereby HDFa cells were seeded into the upper chamber of the CIM-Plate^®^ and relevant conditioned medium taken from control and LMP1-expressing cells in the presence and absence of the TGFβ type II receptor inhibitor, SB431542, and the ERK-MAPK inhibitor, UO126. As shown in Figure 4a inhibition of TGFβ signalling resulted in a significant reduction in cell recruitment when compared to conditioned medium taken from untreated LMP1-expressing cells (*p* < 0.01). Additionally, inhibition of ERK-MAPK signalling also resulted in a significant reduction in HDFa recruitment when compared to conditioned medium taken from untreated LMP1-expressing cells (*p* < 0.001; Figure 4b). No significant difference in recruitment was observed between conditioned medium taken from control cells in the presence or absence of either inhibitor (*p* ≥ 0.05). Significant differences were determined by mixed ANOVA.

### 2.4. Conditioned Medium from LMP1-Expressing Cells Increases Fibroblast Proliferation across Seven Days

When fibroblasts are activated following tissue injury or wounding, they respond by proliferating to support the needs of the healing wound by supplying basal epithelial cells with growth factors to enable repopulation of the wound bed. This biological process is called nemosis and is reviewed by Vaheri et al. [38]. Activated fibroblasts are known as myofibroblasts and are usually short-lived: once the wounded area has been repaired, myofibroblasts undergo apoptosis and are cleared from the site of tissue injury by phagocytosis [39].

**Figure 5:**
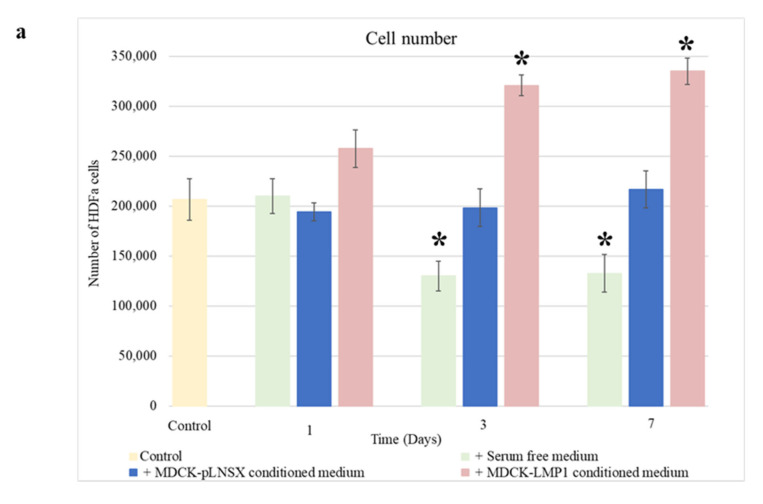

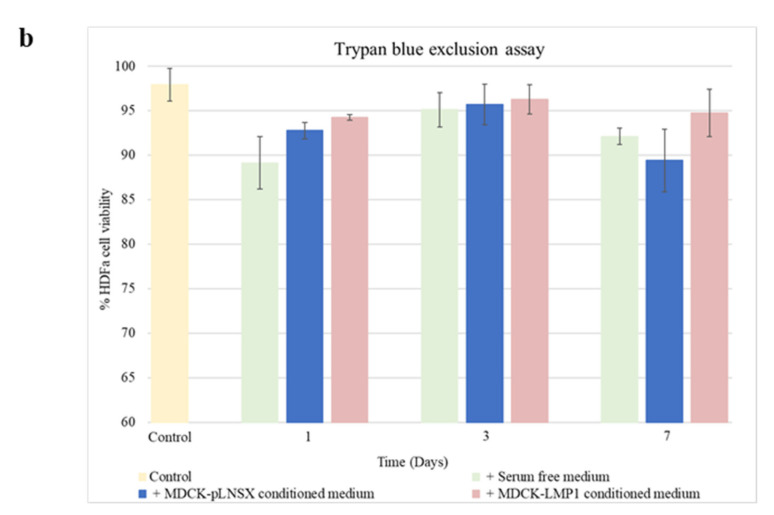
LMP1 enhances HDFa cellular proliferation over the course of a week. (**a**) HDFa cells were cultured in conditioned medium from MDCK-pLNSX control cells, LMP1-expressing cells and serum free medium over a 7-day time course prior to cell counting. The results depict cell number (mean ± SD, *n* = 3). Asterisk indicates significant difference, determined using the Student *t*-test, from day 1 of the same conditions (*p* < 0.01). (**b**) HDFa cells were cultured in conditioned medium from MDCK-pLNSX control cells, LMP1-expressing cells and serum free medium over a 7-day time course prior to trypan blue exclusion assay. The results depict % viable cells (mean ± SD, *n* = 3). Using the Student *t*-test no significant differences were found between conditions (*p* ≥ 0.05).

Having established that conditioned medium from LMP1-expressing cells enhances fibroblast motility and recruitment, and with Figure 1 indicating a slight increase in fibroblast proliferation in response to LMP1-conditioned medium after 3 days, we next sought to establish whether LMP1 was able to stimulate fibroblast activation and therefore enhance fibroblast proliferation and viability across a more extended time period. HDFa cells were cultured in conditioned medium taken from control and LMP1-expressing cells, which were first serum-starved overnight, or with serum-free medium over a 7-day time course before determining cell number using Trypan blue. Figure 5a demonstrates that after 3 days of treatment with LMP1-conditioned medium fibroblast cell count increased significantly (*p* < 0.01) compared to cells treated with conditioned medium from control cells where there was no significant difference (*p* ≥ 0.05). As anticipated, cells cultured in serum-free medium demonstrated a decrease in cell number over the 7-day time course, which is likely due to the lack of growth factors available in the medium. As shown in Figure 5b, none of the three conditions significantly affected cell viability (*p* ≥ 0.05). Significant differences were determined using the Student *t*-test.

### 2.5. Conditioned Medium from LMP1-Expressing Cells Transforms Fibroblasts to Adopt a Myofibroblast-like Phenotype

Activated myofibroblasts facilitate wound closure by secreting cytokines and growth factors, stimulating basal epithelial cell proliferation and migration in order to “close the wound”. According to many recent studies, N-cadherin, E-cadherin, alpha smooth muscle actin (αSMA), vimentin, vinculin and fibronectin are widely accepted as mesenchymal markers in both epithelial-to-mesenchymal transition (EMT) and myofibroblast differentiation [40,41,42,43,44]. Tumours have been described as “wounds that fail to heal” [14], and fibroblasts, including myofibroblasts and CAFs, are known to promote this constitutively active wound response [15].

NPCs featuring a rich infiltrate of CAFs surrounding “nests” of tumour cells indicate poor prognosis [16,17], and LMP1-expressing NPCs are purportedly more aggressive [45,46]. Moreover, LMP1 expression in premalignant lesions results in earlier metastases, potentially linking this viral oncogene to NPC metastasis [47]. In addition, LMP1 has the capacity to transform Rat-1 fibroblasts in vitro [3]; therefore, we sought to discern whether soluble factors secreted by LMP1-expressing epithelial cells could transform fibroblasts in vitro.

It is unclear exactly how long differentiation takes or how long myofibroblasts persist following tissue injury; however, one study identified the myofibroblast phenotype peaked between 5 to 14 days and then decreased between 21 to 28 days post-injury [40]. Another study reported a biphasic pattern of expression of αSMA, a common marker used to identify myofibroblasts, with an increase after 7 days, a decrease at day 14, followed by a significant increase after 20 weeks [48]. By contrast, Sebe et al. [49] observed differentiation after just 3 days. These varying studies all support the inference that differentiation occurs within the first 7 days, therefore based on these findings, we decided to treat HDFas with conditioned medium from control and LMP1-expressing cells and follow fibroblast differentiation over a 72 h period.

**Figure 6:**
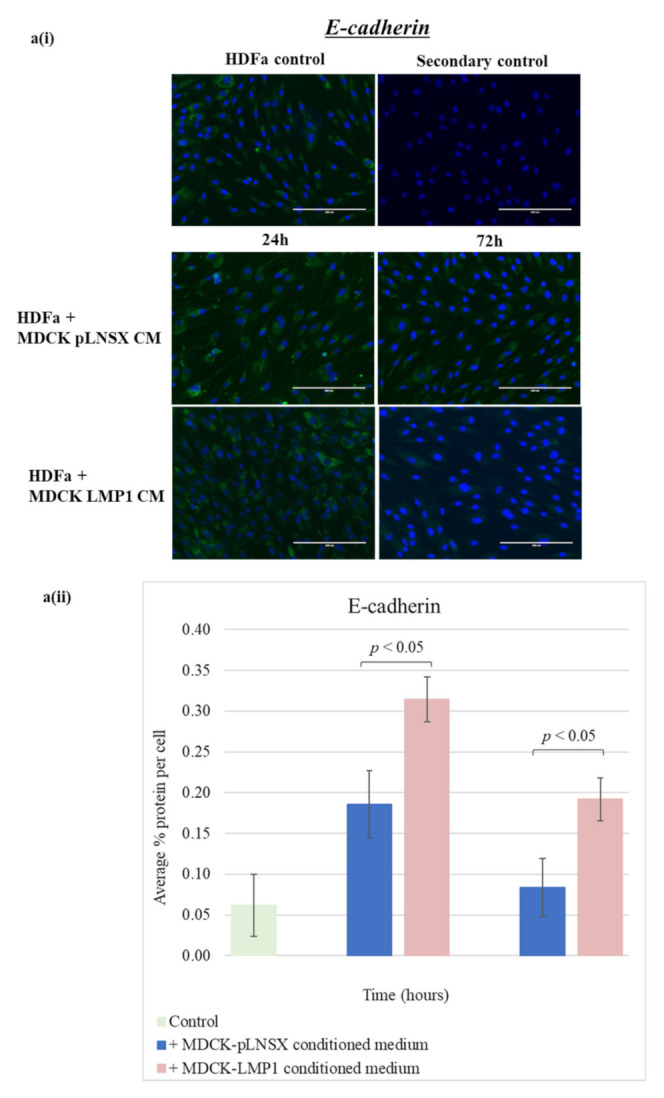

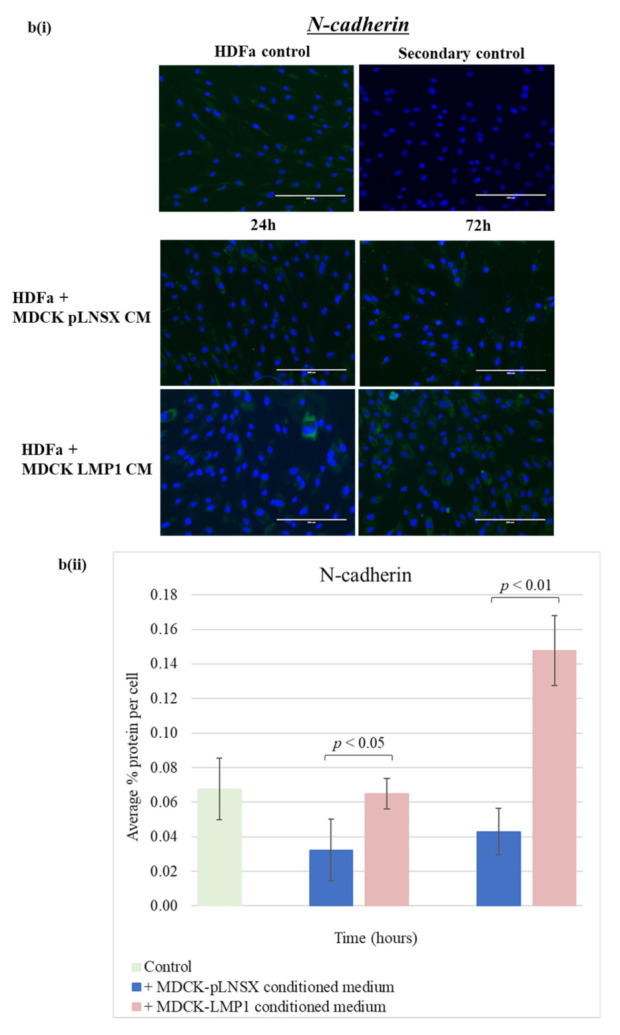

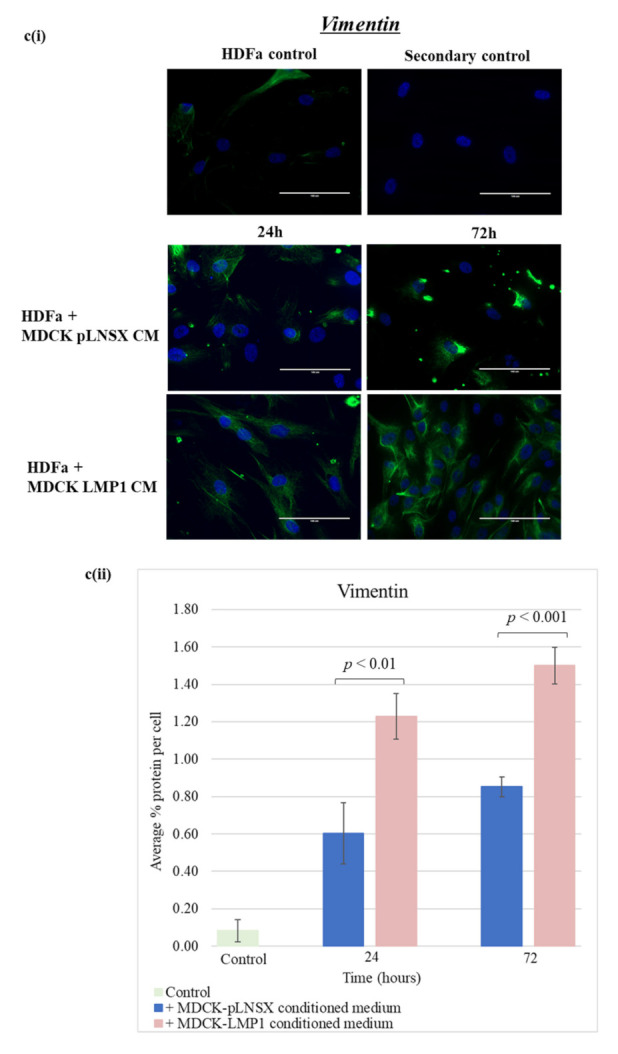

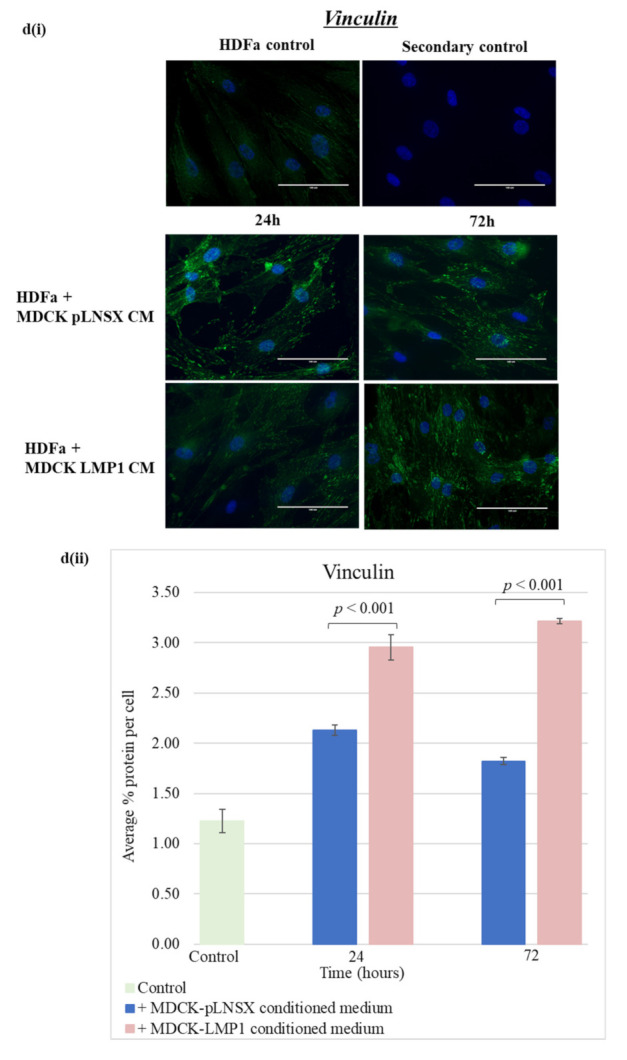

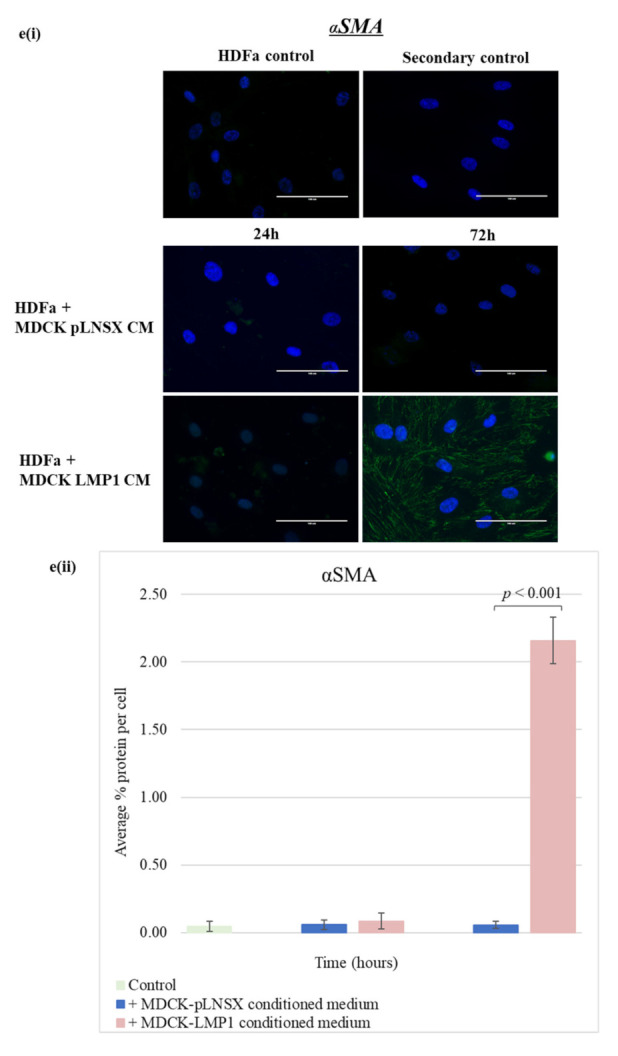

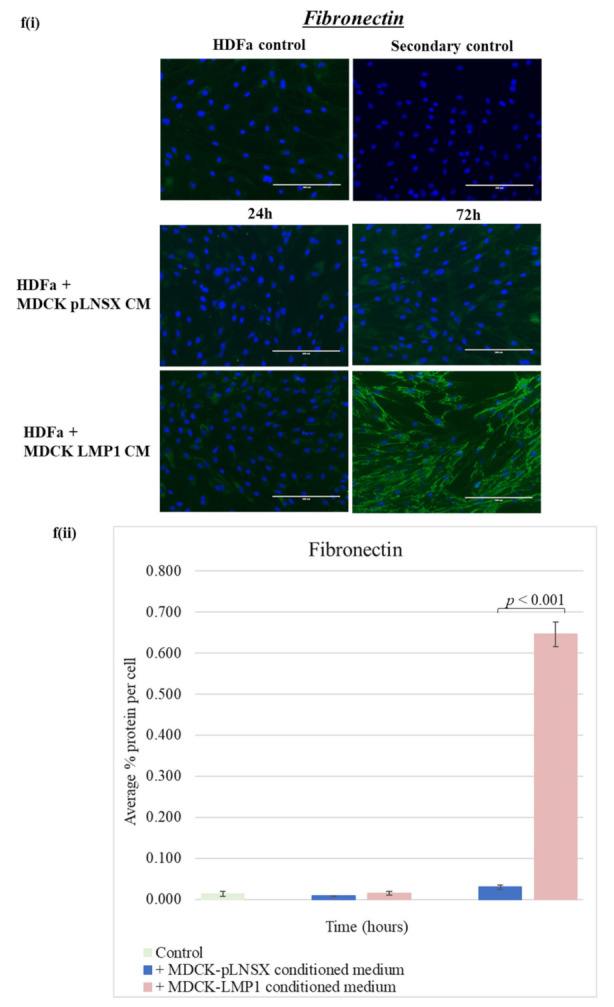
LMP1-conditioned medium alters the expression of myofibroblast markers in human dermal fibroblasts: (**a**) E-cadherin; (**b**) N-cadherin; (**c**) vimentin; (**d**) vinculin; (**e**) alpha smooth muscle actin (αSMA); (**f**) fibronectin. (**i**) HDFa cells were cultured in conditioned medium from control and LMP1-expressing cells over a 72 h time course, prior to immunofluorescence staining for classic markers at set timepoints. All results are representative of experiments performed in triplicate. Bar = 200 μm. (**ii**) Average percentage protein per cell was calculated using ImageJ software (mean ± SD, *n* = 3). All significant differences were determined using the Student *t*-test.

Using immunofluorescence staining, the results demonstrate that after 72 h incubation, LMP1-conditioned medium decreased the expression of E-cadherin (Figure 6(ai)) with concurrent upregulation of the expression of N-cadherin (Figure 6(bi)) in human dermal fibroblasts when compared with human dermal fibroblasts cultured in conditioned medium from control cells. In addition, vimentin and vinculin are also increased in response to LMP1-conditioned medium (Figure 6(ci,di)). Interestingly, there was a marked increase in protein expression observed for the two classic markers of myofibroblasts, αSMA, and fibronectin (Figure 6(ei,fi)). However, staining using the αSMA and fibronectin antibodies did result in a low signal-to-background ratio. Quantification of these observations was carried out using ImageJ software and a colour pixel counter plugin, as shown in Figure 6aii–fii)). Statistical analysis using the Student *t*-test confirmed that LMP1-conditioned medium had a significant effect on the protein expression of all EMT markers that were screened in comparison to conditioned medium from control cells: αSMA, fibronectin, vimentin, and vinculin—*p* < 0.001; E-cadherin—*p* < 0.05; and N-cadherin, *p* < 0.01. Intriguingly, the expression of N-cadherin (Figure 6b), vinculin (Figure 6d) and fibronectin (Figure 6f) in fibroblast cells treated with conditioned medium from control cells also demonstrate an increase after 72 h, while E-cadherin showed a slight decrease (Figure 6a).

### 2.6. Conditioned Medium from LMP1-Expressing Cells Also Transforms Epithelial Cells to Adopt a Mesenchymal-like Phenotype

There is increasing evidence to suggest that in addition to fibroblasts, epithelial cells are also a major source of myofibroblasts in the tumour microenvironment [50]. Therefore, to establish whether the factor(s) in LMP1-conditioned medium could equally transform epithelial cells to adopt a mesenchymal-like phenotype, we repeated the previous experiment with MDCK-pLNSX control cells in place of HDFas.

**Figure 7:**
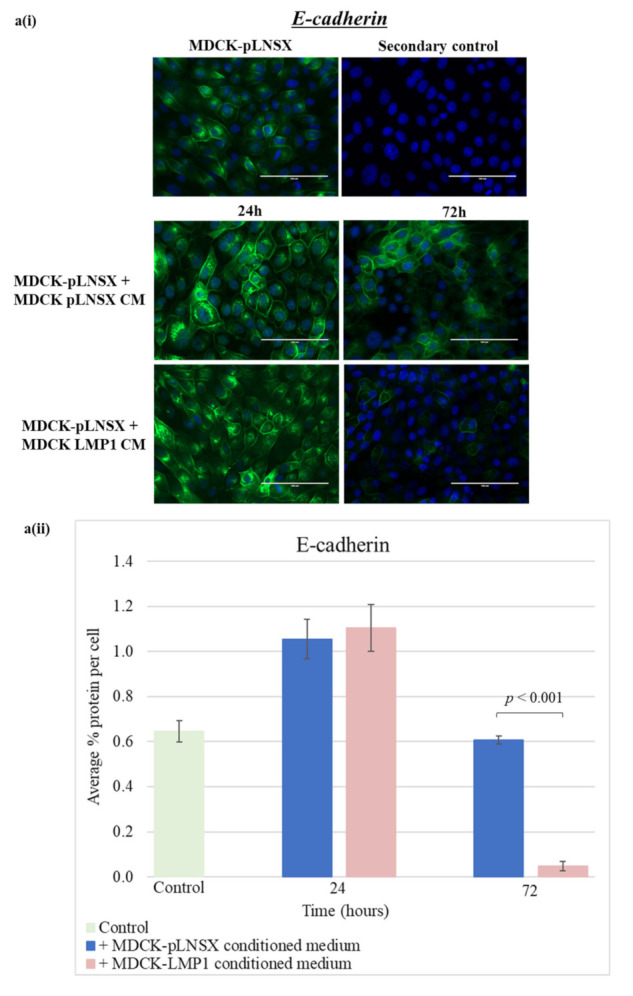

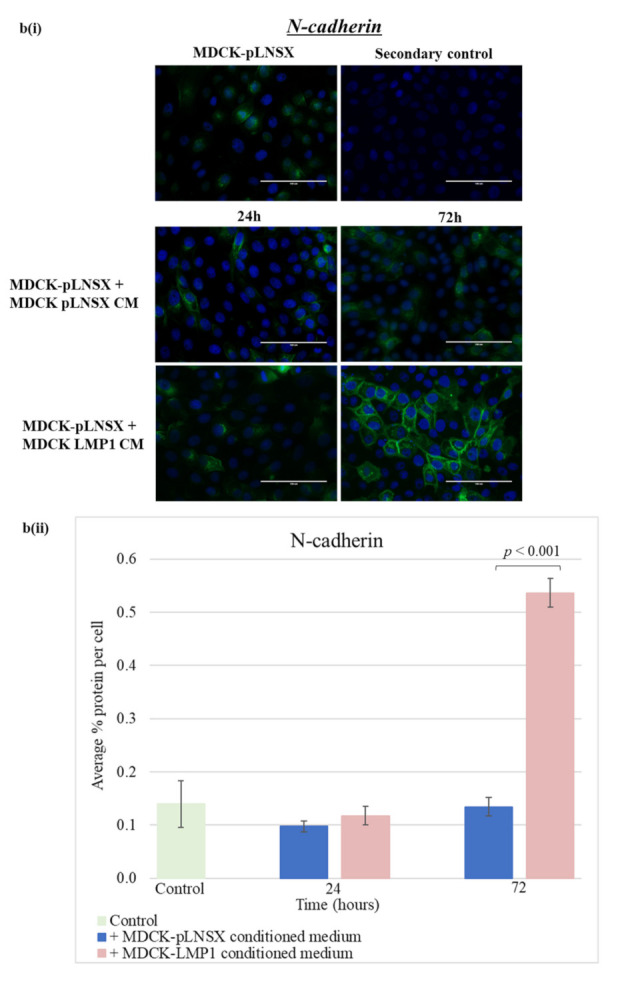

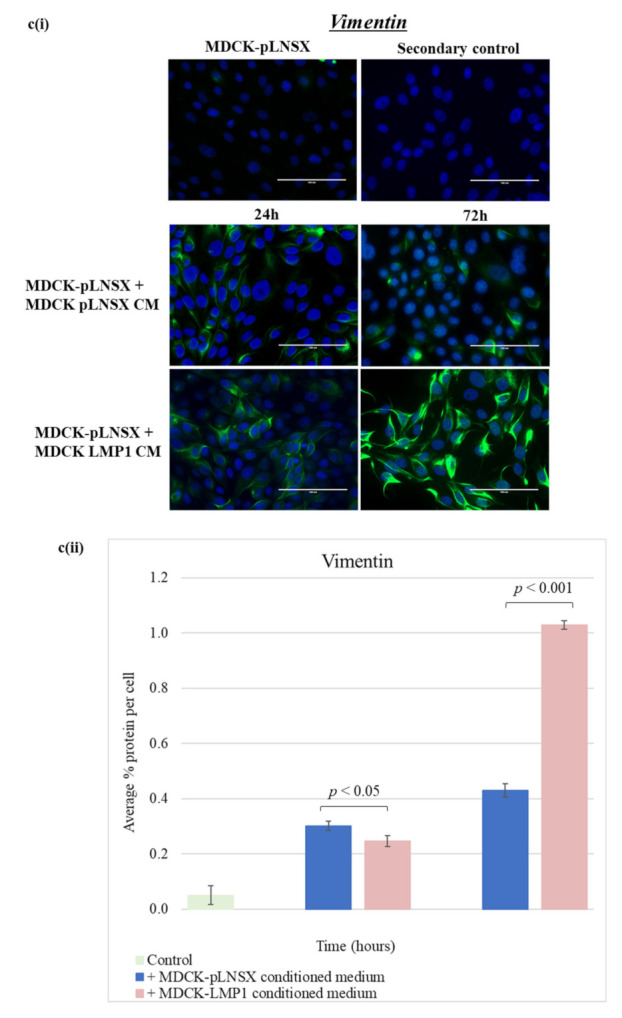

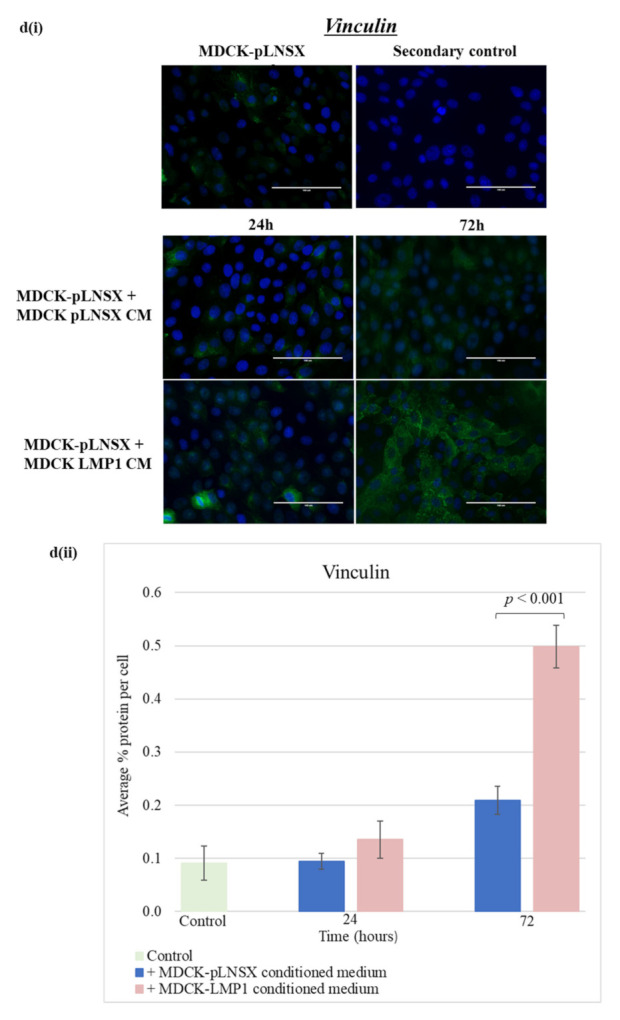

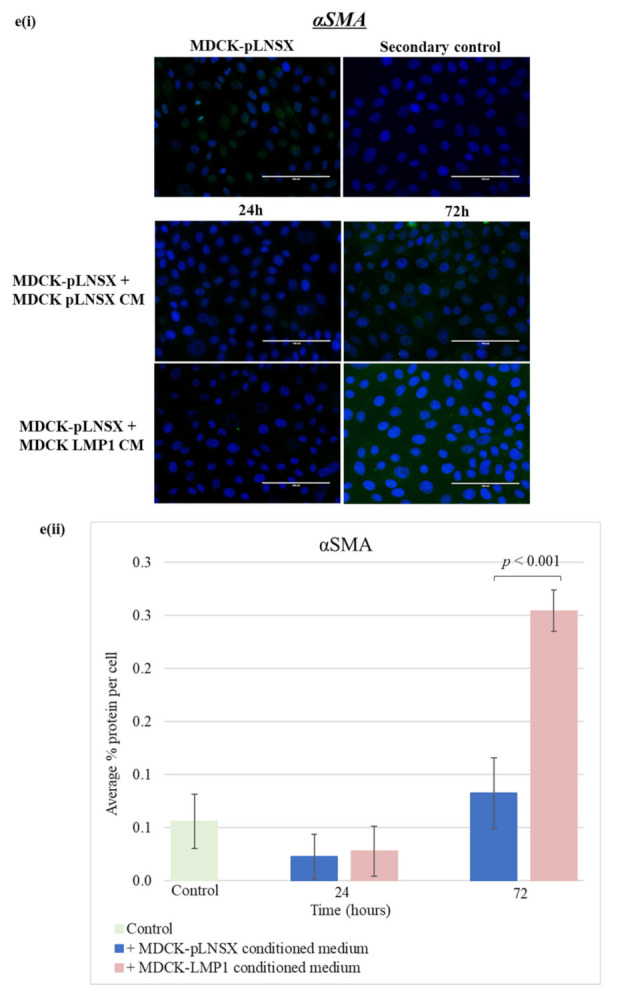

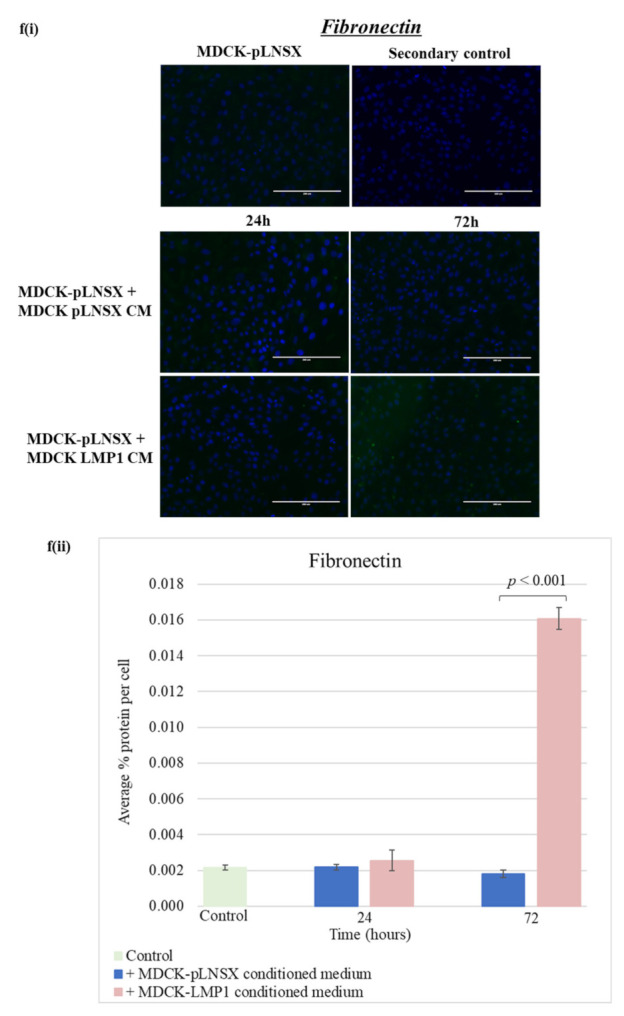
LMP1-conditioned medium alters the expression of EMT markers in epithelial cells: (**a**) E-cadherin; (**b**) N-cadherin; (**c**) vimentin; (**d**) vinculin; (**e**) alpha smooth muscle actin (αSMA); (**f**) fibronectin. (**i**) HDFa cells were cultured in conditioned medium from control and LMP1-expressing cells over a 72 h time course, prior to immunofluorescence staining for classic markers at set timepoints. All results are representative of experiments performed in triplicate. Bar = 200 μm. (**ii**) Average percentage protein per cell was calculated using ImageJ software (mean ± SD, *n* = 3). All significant differences were determined using the Student *t*-test.

Immunofluorescence staining revealed that after 72 h exposure, LMP1-conditioned medium independently decreased the expression of E-cadherin with concurrent upregulation of the expression of N-cadherin in epithelial cells (Figure 7a,b). In addition, vimentin, vinculin and αSMA also demonstrated a marked increase in expression after 72 h treatment with LMP1-conditioned medium (Figure 7c–f). However, staining using the αSMA and fibronectin antibodies once again resulted in low signal-to-background ratios. Quantification of these observations was carried out using ImageJ software and a colour pixel counter plugin, as shown in Figure 7(aii–fii). Statistical analysis using the Student *t*-test confirmed that LMP1-conditioned medium had a very strong significant effect on the protein expression of all EMT markers that were screened (*p* < 0.001). Interestingly, the expression of N-cadherin, vimentin, vinculin and αSMA in epithelial cells treated with conditioned medium from control cells also show a slight increase after 72 h, however this increase is not as dramatic as with LMP1-conditioned medium.

## 3. Discussion

In recent years, a plethora of research has begun to focus on the heterogenous nature of the tumour microenvironment (TME), proposing a role for the non-malignant cells of the TME in tumour progression, invasion and metastasis [51,52]. Of particular interest for this study are carcinoma-associated fibroblasts (CAFs) since emerging evidence suggests an important role for CAFs in the NPC TME. Increased numbers of alpha smooth muscle actin (αSMA)-expressing fibroblasts are found surrounding nests of tumour cells in NPC biopsies, a phenomenon that correlates with shorter overall survival [16,17]. The precise cellular origin and mechanisms leading to CAF activation are largely uncharacterized [38]; however, research suggests that activated fibroblasts/CAFs can originate from multiple cell populations including fibroblasts and epithelial cells via EMT [53,54,55]. LMP1 in extracellular vesicles provide an important contribution to the CAF population in NPC [18], and many of the signalling pathways involved in CAF activation and CAF-driven EMT are also engaged by LMP1, yet the link between LMP1 signalling and CAF formation remains unknown [32]. Malignant cells have been shown to recruit and activate fibroblasts in the TME by increased secretion of TGFβ [21,56,57], whilst LMP1 has been shown to modulate the expression and secretion of TGFβ1, as well as enhance the TGFβ-dependent modulation of ERK/MAPK [9,58].

### 3.1. Conditioned Medium from LMP1-Expressing Cells Enhances Fibroblast Motility, Invasion and Recruitment but Does Not Impact Cell Viability

The presence of CAFs in NPC correlates with poorer prognosis and survival rates [16,17], and LMP1-positive NPCs are reportedly more aggressive [45,46]. Since LMP1 is also known to promote the synthesis, secretion and deposition of fibronectin [9], enhanced migration [30], and can induce an EMT in epithelial cells [7], we wanted to establish whether LMP1 may enhance fibroblast recruitment and alter cell viability.

Qualitative and quantitative analysis confirmed that conditioned medium taken from LMP1-expressing cells enhanced the migration rate of HDFa cells, as measured using the wound healing scratch assay, the transwell migration assay and the xCELLigence^®^ real-time cell assay system (Figure 1a,c,d and Figure 3a,b). Cell proliferation assays confirmed that these effects were not an artefact of cell proliferation but were indeed due to enhanced cell motility across the 3-day period (Figure 1b). Whilst the exact mechanism of fibroblast recruitment is unclear, a number of cytokines have been reported to affect fibroblast motility, including TGFβ1, TGFβ2, platelet-derived growth factor (PDGF) and vascular endothelial growth factor (VEGF) [59,60,61]. Interestingly, gene expression of each of these four cytokines have also been shown to be upregulated by LMP1 [7], and TGFβ1 secretion by LMP1-expressing epithelial cells is also enhanced [9]. Therefore, it is plausible to suggest that LMP1-expressing cells can secrete soluble factors that drive the recruitment of fibroblasts into the TME. Coupled with the findings presented herein and other published findings [18], LMP1 may consequently activate these fibroblasts to become myofibroblasts and ultimately CAFs, that drive oncogenesis in NPC.

Interestingly, whilst LMP1-conditioned medium increases cell count across 7 days, it did not affect epithelial cell or fibroblast viability in this study (Figure 5a,b). This increase in cell count is unsurprising since LMP1 is known to engage a variety of mitogenic signalling pathways, including the ERK/MAPK and p38-MAPK pathways [30,62].

### 3.2. Critical Role for the ERK/MAPK and TGFβ Signalling Pathways in LMP1-Mediated Enhanced Fibroblast Recruitment

There are well-established data supporting the role of TGFβ in enhanced cell motility and invasion [35,36,37]. In addition, a role for the ERK/MAPK signalling pathway in LMP1-induced epithelial cell motility has also been identified [30,33]. Indeed, findings presented herein demonstrate that inhibition of both TGFβ signalling using the TGFβ type I receptor inhibitor, SB431542, which inhibits the Smad-dependent arm of TGFβ signalling and ERK-MAPK signalling using the MEK1/2 inhibitor, UO126, diminish the rate of wound closure and ultimately cell motility and invasion in both control and LMP1-expressing cells, with the latter exhibiting a more significant effect (Figure 3 and Figure 4). These findings suggest that by inhibiting both the Smad-dependent TGFβ and ERK-MAPK signalling pathways independently in LMP1-expressing cells, some of the effects of LMP1-signalling are being blocked, which is, in turn, reducing or inhibiting the secretion of soluble factors into the conditioned medium. However, both these signalling pathways are extremely diverse and complex, including extensive crosstalk with other signalling pathways and regulatory factors, therefore determining the precise mechanism by which this occurs requires further detailed work and investigation.

### 3.3. Conditioned Medium from LMP1-Expressing Cells Induces a Myofibroblast-like and EMT Phenotype in Human Dermal Fibroblasts and MDCK Epithelial Cells, Respectively

In the present study, conditioned medium taken from LMP1-expressing MDCK epithelial cells was shown to modulate the expression of the classic markers of fibroblast activation and EMT, namely E-cadherin, N-cadherin, vinculin, vimentin, αSMA and fibronectin (Figure 6a–f and Figure 7a–f), suggesting a transition from fibroblast to a myofibroblast-like phenotype.

Fibroblast activation is characterised by increased expression of markers such as αSMA, in addition to increased secretion of extracellular matrix (ECM) proteins, including fibronectin [63,64]. In recent years, markers used to identify myofibroblasts and CAFs have become somewhat controversial. A number of studies have found that whilst markers such as αSMA and fibroblast activation protein (FAP) do indicate fibroblast activation, their expression varies amongst activated fibroblasts, and many activated fibroblasts may not express all of these markers at the same time [65]. It is generally agreed that this variety in expression reflects the high degree of heterogeneity of CAFs in the TME, in addition to the dynamic nature of gene expression [54,66].

Whilst cadherin switching from E-cadherin to N-cadherin expression is a classic characteristic of EMT in epithelial cells, it is not usually seen as an indication of myofibroblast differentiation. Indeed, many studies fail to demonstrate E-cadherin expression in fibroblasts [67,68,69]; however, one study did show expression of E-cadherin in fibroblasts, but at a greatly reduced level compared to epithelial cells [70]. In the present study, adult human dermal fibroblasts (HDFa) display a low baseline level of E-cadherin expression, which is lost upon treatment with conditioned medium taken from LMP1-expressing cells, along with a corresponding significant increase in N-cadherin expression (Figure 6a,b). Since N-cadherin is known to be upregulated in myofibroblasts [71], this finding supports the hypothesis that LMP1 is able to induce a transition from a fibroblast to myofibroblast-like phenotype in HDFa cells.

Vimentin is typically expressed in quiescent fibroblasts as it plays a key role in regulating mesenchymal cell shape and motility [72]. Whilst it is true that vimentin expression is upregulated during wound healing [73], it is argued that the effectiveness of vimentin as a CAF-specific marker is greatly hampered by its widespread expression throughout the overall fibroblast population [74]. Nevertheless, increased vimentin expression is associated with enhanced cell motility, a finding which is observed in this current study (Figure 6c). Vinculin is a component of focal adhesions and plays a key role in cell motility [75]: fibroblasts are naturally motile cells and so a higher level of expression of vinculin prior to treatment is to be expected. Following treatment with conditioned medium from LMP1-expressing cells, this small but significant increase in vinculin expression is suggestive of a more motile phenotype (Figure 6d) [76].

In the same vein, LMP1-conditioned medium was also able to induce similar changes in epithelial cells, although to a lesser extent (Figure 7c,d). The distinctive switching of cadherins, characterised by loss of E-cadherin with concomitant upregulation of N-cadherin, is a key feature of cancer-related EMT [42,69]. Additionally, many studies acknowledge an increase in vimentin expression to be a classic hallmark of EMT [77,78,79], an observation that is mirrored in the current study. The increased expression of both αSMA and fibronectin are also known mesenchymal markers of EMT [21,80], and wider research now suggests that myofibroblasts can also originate from other cell populations, for example, epithelial cells [53,54,55]. The findings presented herein demonstrate that conditioned medium from LMP1-expressing cells can mediate the transformation of both epithelial cells and fibroblasts, either of which may conceivably contribute to the formation of CAFs in the NPC TME, however further work is needed to investigate this avenue.

Intriguingly, treatment with conditioned medium from the vector control MDCK epithelial cells also demonstrates a change in protein expression for some of the investigated markers in both epithelial cells and fibroblasts. For example, E-cadherin demonstrates a decrease in expression, whilst N-cadherin and vinculin were seen to increase in both cell lines treated with conditioned medium from control cells (Figure 6a,b,d and Figure 7a,b,d). Additionally, in the epithelial cell line, vimentin expression was increased (Figure 7c), and in the fibroblast cell line, fibronectin expression was increased (Figure 6f). Whilst these changes were observed with conditioned medium from control cells; visually, they were not as profound as the effect of LMP1-conditioned medium. However, further quantitative validation is required to fully assess the extent to which this phenomenon occurs in both epithelial cells and fibroblasts. Moreover, the low signal-to-background ratio observed with both αSMA and fibronectin make it difficult to determine the full extent of this effect and requires further investigation.

Taken together, the characteristic changes in expression of key EMT markers observed in epithelial cells in response to treatment with LMP1-conditioned medium (Figure 7) can be attributed to a more motile and invasive phenotype and are indicative of cells undergoing EMT as previously described in MDCK cells [5,7]. Whilst the characteristic increase in expression of the classic myofibroblast markers αSMA and fibronectin, in addition to the increase in expression of N-cadherin, vimentin and vinculin in fibroblasts following treatment with LMP1-conditioned medium (Figure 6), suggests activation of the fibroblasts into a myofibroblast-like phenotype. These findings support the concept that conditioned medium from LMP1-expressing cells induces EMT in MDCK epithelial cells and fibroblast-to-myofibroblast differentiation in human dermal fibroblasts, and therefore, it is plausible that LMP1 may transform multiple cell types within the local tumour milieu to facilitate the growth, invasion and migration of NPC tumour cells.

## 4. Materials and Methods

### 4.1. Cell Lines

The canine kidney epithelial cell line, MDCK, has been described previously [81]. MDCK cells expressing the neomycin resistance gene (neoR) and wildtype LMP1 have been described previously [82]. The adult human dermal fibroblasts (HDFas) were purchased from (Thermo Fisher Scientific, Paisley, UK). All cell lines were cultured in high glucose DMEM (Thermo Fisher Scientific, Paisley, UK) supplemented with 10% fetal calf serum (FCS), 1% antibiotic/antimycotic solution (HyClone Thermo Fisher Scientific, Paisley, UK), and for the MDCK cell lines only, G418 (Invitrogen; 400 μg/mL) to select for cells containing the neomycin resistance cassette.

### 4.2. Conditioned Medium

Stock flasks of MDCK-pLNSX and MDCK-LMP1 were cultured and maintained as described in Section 4.1. To obtain conditioned medium for treating HDFa cells, each stock flask was serum-starved for 24 h using true serum-free DMEM (0% FCS, 1% antibiotic/antimycotic solution). After 24 h, conditioned medium was harvested, centrifuged for 10 min at 300× *g* to remove cell debris and used to culture the HDFa and MDCK-pLNSX control cells for the time-course experiments in Figure 6 and Figure 7. Stock flasks were serum-starved and conditioned medium harvested twice a week. To obtain conditioned medium for the chemical inhibitor experiments in Figure 3 and Figure 4, a separate stock plate of MDCK-pLNSX and MDCK-LMP1 cells was maintained, serum-starved and treated with the relevant pharmacological inhibitors (see Section 4.3) and incubated for 24 h as before. After 24 h, the inhibitor-treated conditioned medium was harvested and used to culture the HDFA and MDCK-pLNSX control cells for the experiments in Figure 3 and Figure 4.

### 4.3. Treatment with Selective Pharmacological Inhibitors

Cells were seeded at 1 × 10^6^ cells/plate in 10cm plates and allowed to adhere overnight in complete growth medium. Prior to treatment, cells were washed twice with pre-warmed PBS to remove any residual serum and cytokines present, then incubated in medium containing 0.5% FCS containing the appropriate concentration of specific pharmacological inhibitors based on our previous studies [7,9], as outlined in Table 1, or using the carrier solvent DMSO as a control, and incubated overnight prior to downstream experiments.

### 4.4. Wound Healing Scratch Assay

Cells were seeded at 2 × 10^5^ cells/well of a 6-well plate and grown to confluency. Prior to treatment, cells were “serum-starved” overnight by incubation in media containing 0.5% FCS. The following morning, the confluent monolayer was scratched using a disposable yellow pipette tip and the cells allowed to recover in the incubator for 30 min. Once recovered, the cells were washed and treated with relevant pharmacological inhibitors or carrier solvent, as described in Section 4.3, and a position for photographing was marked on the underside of the plate for each well. Wound healing was monitored by imaging the same position at various timepoints using the EVOS^®^ FL Cell Imaging System (Life Technologies, UK). The rate of wound closure was measuring using ImageJ software to measure the area of the wound (see Section 4.10.2). Plates were returned to the incubator between photographing.

### 4.5. Cell Proliferation Assay

Cells were seeded at 1 × 10^4^ cells/well in 96-well plates in 200 μL medium and left to adhere overnight. The following afternoon, media was replaced with media containing 0.5% FCS and incubated overnight once more. After each timepoint, cell proliferation assays were carried out using the Promega CellTiter 96^®^ Aqueous One Solution Cell Proliferation Assay kit (Promega, Southampton, UK) following the manufacturer’s instructions. Spent media was removed from all wells, followed by addition of 100 μL of 0.5% FCS-containing media and 20 μL of CellTiter 96^®^ Aqueous One Solution Reagent. The plate was then incubated at 37 °C with 5% CO_2_ for 4 h. Absorbance was read at 490nm using a GloMax^®^ Discover Microplate Reader (Promega, UK).

### 4.6. Transwell Migration Assay

Serum-starved cells were recovered as single-cell suspensions, and 2.5 × 10^4^ cells were seeded into the upper well of a transwell migration chamber (Corning) coated with fibronectin (10 μg/mL in sterile PBS). After 16 h of incubation at 37 °C, the wells were fixed in 30% ice cold methanol and stained with 1% crystal violet (in sterile distilled water). The percentage of cell migration was determined after photographing representative fields and counting the number of stained (migrating) cells. The extent of cell migration was quantified using ImageJ software by calculating percentage surface area covered by the migrating (stained) cells (see Section 4.10.1).

### 4.7. RCTA xCELLigence Real-Time Assay

Cells were seeded at 3 × 10^4^ cell/well in the upper chamber of a cell invasion/migration plate (CIM-Plate^®^). Relevant control or conditioned medium was added to the bottom chamber of the CIM-Plate^®^ and the plates were placed in the xCELLigence real-time cell assay dual purpose (RTCA-DP) system (Agilent Technologies LDA UK Ltd, Stockport, UK) prior to incubating the whole system at 37 °C with 5% CO_2_ for 48 h. Cell index was automatically calculated from surface area coverage using the ACEA real-time cell analysis software.

### 4.8. Immunofluorescence Microscopy

Cells grown on Teflon-coated microscope slides (Hendley-Essex, Loughton, UK) were processed for imaging as described previously, with the exception of the blocking agent used here (1% BSA) [82]. Samples were incubated with primary antibodies followed by secondary antibodies in 1% BSA. A complete list of antibodies is outlined in Table 2. Slides were mounted with coverslips using a few drops of glycerol mounting agent containing 4′,6-diamidino-2-phenylindole (DAPI) and 1,4-diazabicyclo-[2,2,2]-octane (DABCO; Abcam, UK). Images were obtained using the EVOS^®^ FL Cell Imaging System (Thermo Fisher Scientific, Paisley, UK) and processed using ImageJ (see Section 4.10.1).

### 4.9. Trypan Blue Exclusion Assay

Cells were recovered as single cell suspensions and centrifuged at 300× *g* for 5 min. Cell pellets were resuspended in 100 μL of media, and 50 μL of cell suspension was placed into an Eppendorf with an equal volume of 0.4% trypan blue dye (Sigma-Aldrich, Gillingham, UK) prior to mixing by gentle pipetting. The mixture was incubated for 3 min at room temperature, followed by cell counting using a Fast-Read single use counting chamber slide (Immune Systems, Paignton, UK). Clear and blue stained cells were counted in each large punnett square of the counting chamber. Percentage viable cells was determined by dividing the number of viable (clear) cells by the number of total cells and multiplying by 100.

### 4.10. Quantification Analysis Using ImageJ Software

#### 4.10.1. Colour Pixel Counter Plugin

The colour pixel counter plugin was used to quantify protein expression in Figure 6 and Figure 7 and migration via the transwell migration assay in Figure 1c. After selecting the colour of interest, number of pixels was automatically calculated.

#### 4.10.2. Wound Area in WHSA

First, the scale was set to a known distance in μm using the scale bar on each image. The area of the wound was then measured by using the freehand tool to trace the wound and selecting “measure”. Further calculations including % wound closure were then calculated using Microsoft Excel.

### 4.11. Statistical Analyses

Numerical data was analysed by Student *t*-test, one-way analysis of variance (ANOVA) or mixed (ANOVA). Specific details are given in the figure legends. Microsoft Excel was used to perform the Student *t*-test when determining the significance of the difference between the means of two sets of data. IBM SPSS Statistics 23 (IBM Corporation, New York, NY, USA) was used to perform all ANOVAs. *p* values < 0.05 were considered statistically significant. On figures where *n* = 3 is stated, experiments were performed in technical triplicate on three separate occasions.

## 5. Conclusions

### Summary and Future Work

It is widely accepted that LMP1 is the major transforming oncoprotein encoded by EBV and is known for its ability to transform a number of cell types in addition to inducing an EMT in MDCK epithelial cells. Findings presented herein demonstrate that conditioned medium from cells expressing LMP1, which may contain a soluble factor, or factors, secreted by these LMP1-expressing cells, enhances the transformation, cell motility, invasion and recruitment of both fibroblasts and epithelial cells in vitro, indicating a pivotal role for such soluble factors in contributing to the highly invasive and metastatic nature of the TME.

At this stage, these soluble factors remain unidentified. Based on current research, TGFβ is a viable candidate implicated in the phenomena observed in the present study. However, LMP1 is known to upregulate many cytokines and chemokines as well as ECM proteins, including fibronectin, that may also play a key role. It is plausible, if not probable that more than one factor is responsible for these observations, and there may be synergistic interplay between multiple cytokines, chemokines and ECM proteins, for which further investigation is required to define these factors formally.

Additionally, the effects of enhanced motility, invasion and recruitment observed herein are diminished by both ERK-MAPK, and to a lesser extent, TGFβ inhibition. Smad-dependent TGFβ signalling is required for the processes involved in cellular differentiation [82], suggesting that inhibition of this pathway may block differentiation processes, such as EMT. However, in a recent study, we demonstrated no role for TGFβ signalling in generating an EMT in MDCK epithelial cells [7]. Whether the small molecule inhibitor, SB431542, can attenuate Smad-independent signalling remains unclear, and previous studies have suggested this is not the case [9]. The results shown in Figure 3 and Figure 4 showed that blocking TGFβ signalling diminished cell motility, but not as profoundly as blocking the ERK-MAPK pathway. These findings support well-established data demonstrating that the TGFβ signalling pathway, particularly the non-Smad arm, participates in crosstalk with the ERK-MAPK pathway, and so it would be pertinent to further unpick the role of the non-Smad arm of the TGFβ signalling pathway in LMP1-mediated phenomena such as these.

As previously mentioned, CAFs can originate from various different cell populations, including transformed fibroblasts and epithelial cells having undergone EMT [39]. Since the current study demonstrates that treatment with LMP1-conditioned medium induces expression of the classic myofibroblast markers, αSMA and fibronectin, it is plausible that LMP1-mediated transformation of either fibroblasts or epithelial cells may contribute to CAF formation in NPC. It would be interesting to conduct lineage tracing experiments in CAFs derived from NPC biopsies, for example, by analysing the expression of additional CAF markers such as fibroblast activation protein (FAP) from different microenvironment niches.

**Figure 8:**
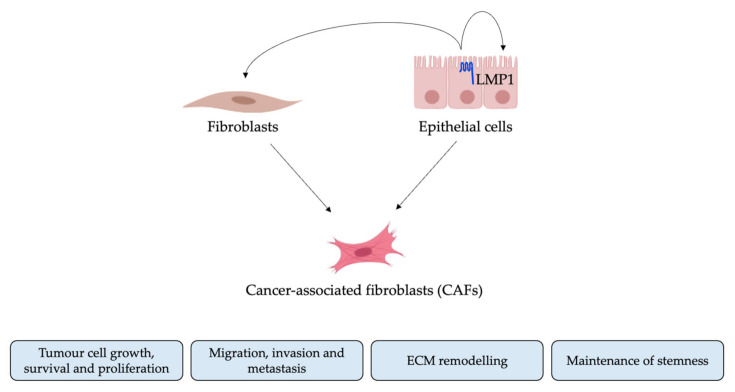
Potential effects of LMP1′s contribution to CAFs in the NPC tumour microenvironment. CAFs can originate from multiple potential cell sources, including normal fibroblasts and epithelial cells via EMT. Once activated, CAFs are able to mediate several processes that promote tumour progression. LMP1 may stimulate fibroblast-to-myofibroblast differentiation via paracrine signalling, or induce autocrine stimulation of epithelial-to-mesenchymal transition in host cells.

In summary, the results presented in this current study demonstrate that conditioned medium taken from LMP1-expressing cells induce EMT in epithelial cells and activates fibroblast-to-myofibroblast differentiation in human dermal fibroblasts. Furthermore, conditioned medium from LMP1-expressing cells enhances cell motility and invasion in both epithelial cells and fibroblasts—an effect that is abrogated by inhibition of both TGFβ and ERK-MAPK signalling (Figure 8). Moreover, results presented herein demonstrate that fibroblast recruitment is enhanced by LMP1-conditioned medium. This evidence provides important insights and a promising foundation upon which to direct further research into the role of LMP1 in modulating the TME in NPC. Establishing and understanding the mechanisms by which LMP1 contributes to the TME and tumourigenesis in NPC could provide potential new therapeutic targets for cancer treatments.

## Figures and Tables

**Table 1:** Summary of small molecule inhibitors and carrier solvent concentrations used.

Inhibitor/ Carrier Solution	Stock Concentration	Working Concentration	Supplier (Cat. No)	Pathway Modulated
SB431542	10mM (in DMSO)	25 μM	Tocris (1614)	Inhibitor of TGFβRI
UO126	10mM (in DMSO)	10 μM	Tocris (1144)	Inhibitor of MEK1/2
DMSO	-	1:1000	Thermo Fisher Scientific (10103843)	Inert carrier solvent

**Table 2:** Summary of antibodies used for immunofluorescence staining.

Antibody	Species	Dilution (with 1% BSA)	Target	Supplier (Cat. No)
**Primary antibodies**				
Anti-E-cadherin	Mouse	1:100	E-cadherin	BD Biosciences (610182)
Anti-N-cadherin	Mouse	1:100	N-cadherin	Abcam (ab124397)
Anti-Vimentin	Mouse	1:200	Vimentin	Thermo Fisher Scientific (OMA1-06001)
Anti-Vinculin	Mouse	1:200	Vinculin	Sigma-Aldrich (V9131)
Anti-αSMA	Mouse	1:100	αSMA	Abcam (ab7817)
Anti-Fibronectin	Mouse	1:100	Fibronectin	Thermo Fisher Scientific (MA5-11981)
**Secondary antibodies**				
Anti-mouse	Goat	1:1000	Mouse	Thermo Fisher Scientific (A11001)

## Data Availability

Data is contained within the article.

## Abbreviations

αSMA	Alpha smooth-muscle actin
ANOVA	Analysis of variances
CAF	Cancer-associated fibroblast
CIM	Cell invasion and metastasis
EMT	Epithelial-to-mesenchymal transition
EBV	Epstein–Barr virus
ECM	Extracellular matrix
ERK-MAPK	Extracellular signal-regulated kinases—mitogen-activated protein kinases
FAP	Fibroblast activation protein
HDFa	Adult human dermal fibroblast
JNK/SAPK	Jun N-terminal kinase—stress-activated protein kinase
LMP1	Latent membrane protein 1
MDCK	Madin-Darby canine kidney epithelial cell
MSC	Mesenchymal stem cells
NPC	Nasopharyngeal carcinoma
NK cell	Natural killer cell
NF-κB	Nuclear factor kappa-light-chain-enhancer of activated B cells
p38-MAPK	Mammalian p38 mitogen-activated protein kinase
PI3K	Phosphatidylinositol 3-kinase
PDGF	Platelet-derived growth factor
RTCA-DP	Real-time cell assay dual purpose
TGFβ	Transforming growth factor beta
TME	Tumour microenvironment
VEGF	Vascular endothelial growth factor
WHSA	Wound healing scratch assay
